# Providers’ perspectives of the neonatal intensive care unit context and care provision for adolescent parents: an interpretive description

**DOI:** 10.1186/s12884-023-05553-1

**Published:** 2023-04-17

**Authors:** Elizabeth Orr, Marilyn Ballantyne, Andrea Gonzalez, Susan Michelle Jack

**Affiliations:** 1 Department of Nursing, Brock University, Faculty of Applied Health Sciences, 1812 Sir Isaac Brock Way, St. Catharines, Ontario L2S 3A1 Canada; 2 Holland Bloorview Kids Rehabilitation Hospital, Toronto, Ontario Canada; 3 Department of Psychiatry & Behavioural Neurosciences, McMaster University, Hamilton, Ontario Canada; 4 McMaster University, School of Nursing, Hamilton, Ontario Canada

**Keywords:** Adolescent parents, Intensive care units, Neonatal, Qualitative interpretive methods, Nurses, Nursing, Stigma

## Abstract

**Background:**

The neonatal intensive care unit (NICU) is a complex care environment, with the NICU patient population among the most vulnerable in a hospital setting. Adolescent parents are a unique group within the broader NICU parent population and admission of their infant to the NICU contributes to an already complex situation as adolescent pregnancy and parenting is often associated with a range of psychosocial challenges. How the NICU care context influences care provision for adolescent parents is a significant gap in the NICU parenting and support discourse. Therefore, this study aimed to explore health and social care providers’ perspectives of the NICU care context and how providers perceive the context as influencing the experiences of adolescent parents in the NICU.

**Methods:**

This was a qualitative, interpretive description study design. In-depth interviews were conducted with providers, including nurses and social workers, caring for adolescent parents in the NICU. Data was collected between December 2019 and November 2020. Data were analyzed concurrently with data collection. Constant comparison, analytic memos, and iterative diagramming techniques were used to challenge developing analytic patterns.

**Results:**

Providers (*n* = 23) described how the unit context influenced care provision as well as experiences for adolescent parents. We learned that having a baby in the NICU was perceived by providers as a traumatic experience for parents – impacting attachment, parenting confidence and competence, and mental health. Environmental factors – such as privacy and time – and perceptions that adolescent parents are treated differently in the NICU were also seen as influencing this overall experience.

**Conclusions:**

Providers involved in the care of adolescent parents in the neonatal intensive care unit described the distinctiveness of this group within the broader parent population and how quality of care may be impacted by contextual factors as well as experiences of age-related stigma. Further understanding of NICU experiences from the parents’ perspectives are warranted. Findings highlight opportunities for strengthened interprofessional collaboration and trauma- and violence-informed care strategies within the neonatal intensive care environment to mitigate the potential negative influence of this experience and improve care for adolescent parents.

## Introduction

The neonatal intensive care unit (NICU) is an intricate and multifaceted care environment, with the NICU patient population among the most vulnerable in a hospital setting. In addition to attending to the complex biophysical and developmental needs of neonatal patients, care providers in the NICU are also tasked with supporting the social-emotional needs of parents and family members using a model of family centred care (FCC) as a guide [1–3]. Parents in the NICU are at an increased risk for depression, anxiety, acute stress, and posttraumatic stress disorder – with ill-effects of the NICU experience lasting well-beyond the discharge of the infant [4, 5]. This parental impact has led to an increased focus on understanding parental experiences of the NICU [6, 7], as well as interventions to mitigate associated adverse outcomes [8].

Adolescent parents (less than 20 years of age) are a unique group within the broader NICU parent population [9]. Infants born to adolescents are at an increased risk for preterm birth, low birth weight, and congenital anomalies, making these infants more likely to require hospitalization in a NICU shortly after birth [10, 11]. For adolescent parents, admission of their infant to the NICU contributes to an already complex situation as adolescent pregnancy and parenting is often associated with a range of health and psychosocial challenges, for example, risk factors associated with adolescent pregnancy include low socio-economic status [12], adverse childhood experiences (ACEs) and childhood disadvantage [13, 14], mental health challenges [15], and exposure to violence [12]. In addition, health harming behaviours such as smoking and substance use are higher among pregnant adolescents [11]; and behaviours seen as protective or preventive are lower among adolescents, including antenatal care rates [16] and breastfeeding [17]. Many of these risks are also confounded by experiences of inequity grounded in the social determinants of health [18]. Despite a relatively good understanding of general parental experiences of the NICU, adolescent parents remain an under-explored sub-group of the NICU parent population [19]. Moreover, how the NICU care context influences care provision for adolescent parents and the perceived impact on their NICU experience is a significant gap in the NICU parenting and support discourse. For this study, a relational view of the NICU care context was adopted [20]. When considering context in a relational way, structures and dynamic processes are seen as constantly shaping and being shaped by people’s lives. Therefore, how people influence and are influenced by any given context can have implications for their health and wellbeing [20]. The NICU care context for this study is defined as the complex structures and processes (e.g., environment, policy, roles, relationships) shaping and shaped by the individuals engaging in this context (e.g., parents, staff, visitors). The purpose of this paper is to present findings of a qualitative interpretive description study exploring providers’ perspectives of how the NICU care context influences the care that is provided to adolescent parents as well as the providers’ perspectives of the NICU experience for adolescent parents.

## Background

### Parents’ experiences of the NICU

Parents in the NICU – in addition to experiencing the critical illness and uncertainty related to long-term outcomes for their infant – often face emotional and psychological consequences related to this hospitalization. A recent meta-analysis reported parental stress in the NICU as a worldwide healthcare issue [5]; with alterations in the parental role cited as the greatest source of stress. This stress was found to be independent of the infants’ health risk – meaning the NICU hospitalization itself was a source of parental stress regardless of the degree of illness of the infant. The emotional and psychological outcomes experienced by parents are pervasive and continue well beyond the infant’s discharge from the NICU [21]; with studies investigating the prevalence and longevity of these symptoms reporting ill-effects up to seven years post-discharge [4].

While the experiences of adult-aged parents in the NICU are well documented, the experiences of the adolescent parents within the context of the NICU are poorly understood. In a review of the literature on adolescent parenting in the NICU, the majority of the research focused on parental stress and parenting practices among adolescent parents with infants in the NICU (with adolescent parents often only a sub-population within the study sample) and a few studies examining other aspects of the NICU experience, including communication practices, parental needs, and evaluation of various intervention programs [19]. Since the time of this review, studies that specifically focus on adolescent parents with infants in the NICU remain scarce but underscore the importance of further understanding with this parent population [9]. What we have learned from literature outside of the NICU context is that adolescent parents often experience health care differently than their adult counterparts and this is discussed in the following section.

### Adolescent parents’ health care experiences

In general, adolescents are a relatively healthy population with decreased health care utilization during the teen years [22]. When they do access the health care system, adolescents are cautious about revealing sensitive information for fear of judgement and are particularly concerned about privacy and confidentiality. Pregnant and parenting adolescents require enhanced engagement with the health care system compared to their non-pregnant counterparts as well as tailoring of services to meet their unique needs (e.g., healthy growth and development, mental health) and mitigate associated risks [22]. However, stigma surrounding adolescent parenting and judgemental attitudes of health care providers toward pregnant adolescents can contravene this engagement [9, 23].

Adolescent parents report negative health care encounters associated with judgemental attitudes by providers toward adolescent pregnancy [22]. An example of this type of negative experience or judgement is the presumption of substance use by care providers, leading to fear of involvement of child protection services. In contrast, features of positive experiences included respectful, supportive, and non-judgemental care [22]. Labour, birth, and the immediate postpartum period are critical points of contact with the health care system for adolescent parents. Nursing care during this time can leave a young person feeling respected and confident in the parenting role; or judged, disempowered and vulnerable [23]. While experiences during the postpartum period vary, negative experiences are cited as more memorable and hurtful [24]. Despite encountering adolescent parents during this vulnerable period, perinatal nurses may lack education specific to the care of adolescents and knowledge of hospital- and community-based resources for adolescent parents [25]. When adolescent parents have negative health care experiences related to the above mentioned factors (e.g., stigma, lack of trust, or in adequate programming) the health care and social care services designed to improve parent and child health outcomes might in fact be difficult for adolescent parents to access and engage with leading to inequities.

### The NICU care context

The NICU is a complex context to begin a parenting journey, with the above-mentioned negative impact on emotional wellbeing serving to underscore the influence of this experience on individuals’ and families’ lives. Parents cite several contextual factors as contributing to their experiences in the NICU (both positive and negative) including logistical factors such as transportation, housing/accommodation close to the hospital, and access to affordable and nutritious food; relational factors such as trusting relationships with care providers, continuity of care (e.g., availability of primary nurses and physicians), unbiased/non-judgemental care, and perceived time for parental questions/concerns; and social-emotional factors such as flexible visiting polices with limited restriction on access to infant, availability of parent-friendly spaces, and access to targeted supports [26–29]. Despite various interventions implemented and evaluated to mitigate the negative impact of the NICU environment on parental experiences, especially in attempts to reduce parental stress or improve coping [8], tailored interventions for adolescent parents are scarce and the demographics of parents participating in evaluated interventions are often distinctly different from the adolescent parent population (e.g., mean participant age, marital status and education) [30, 31].

NICU providers also share insights into some of the factors related to the NICU care context that can influence parental experiences. For example, the high stress atmosphere of the NICU was viewed as challenging for the implementation of family centred care (FCC) principles (e.g., information sharing, respect and honoring differences, partnership and collaboration) [32]. Additionally, NICU environmental characteristics, such as staffing and resource adequacy, nurse-physician relationships, and nurse-management relationships, were described as impacting nurse-rated quality of care and levels of care rationing [33]. Research findings also suggest that NICU patients and families may go without parental support and care coordination to maintain technologically oriented care, life-support, and surveillance tasks when less favourable work environments are reported [32, 33]. These perspectives into the NICU care context and its potential impact on how care is provided and experienced are important, however, lacking an adolescent focus there remains significant gaps in understanding how best to support and care for these families within the NICU.

## Methods

### Aims

Adolescent parents and their infants experience many complexities and vulnerabilities. This may be exacerbated when the infant of an adolescent parent is admitted to the NICU shortly after birth. In this situation, the risk factors associated with adolescent pregnancy and parenthood intersect with the pervasive emotional and psychological effects of the NICU experience. However, little is known about this intersection, rendering health and social care providers ill-equipped to support these families within the NICU and a post-discharge to promote optimal growth and development of parent and infant. Therefore, the aim of this study was to conduct and in-depth analysis of the NICU care context from the providers’ perspective including how providers perceive the context influencing care provided to adolescent parents in the NICU.

### Research question

The research question guiding this study was: what can be learned about the NICU care context from the providers perspective and how do providers perceive this context as influencing the experiences of adolescent parents in the NICU?

### Design

Interpretive description was used to guide the methodological decisions this study. Interpretive description is an interpretive/constructivist, applied qualitative research approach that aligns with the disciplinary knowledge requirements of nursing and other applied health professions in that it emphasizes research questions that are clinically relevant and findings that are application ready [34]. Interpretive description design does not require the use of existing theoretical frameworks for the positioning of the research work but instead encourages consideration of the knowledge needs of the intended audience to guide the design logic of the project [35]. A nursing disciplinary orientation – including a holistic view of individual, family, and community health as well as multiple ways of knowing – contributed to the scaffolding of this study, from framing the research question to informing participant recruitment, data collection and analysis [36]. This research approach also supports the collection of data from provider participants, highlighting the role of thoughtful clinicians as rich sources of qualitative data for applied health research [34].

### Participants

Providers are a rich source of qualitative data in applied health research [34]. This study capitalizes on the fact that provider-participants see many instances of a phenomenon over time – in this case providing care to adolescent parents in the NICU – and are therefore well positioned to spot variations and speak to patterns that may not be realized in interviews with parents alone.

A purposeful sample of health care and social care providers involved in therapeutic relationships with adolescent parents in the NICU were recruited. Providers were included if they self-identified as having experience caring for adolescent parents who have had their infant in the NICU. Broad criteria for inclusion allowed for maximal variation and a range of perspectives based on discipline, role, and practice setting. Team leaders and/or managers at seven sites across southern Ontario, Canada acted as recruitment partners; these sites included four NICUs and three public health units (PHUs). Public health units were valuable recruitment partners because in the context of where this study was conducted (Ontario, Canada) PHUs are responsible for delivering the Nurse-Family Partnership® (NFP) program. Nurse-Family Partnership is a targeted public health intervention aimed at improving maternal-child health outcomes for young pregnant and parenting adolescent girls and young women through nurse home visiting [37] and PHNs delivering this program often supported their adolescent parent clients before, during, and after NICU admission.

Recruitment partners assisted the research team by distributing recruitment emails to relevant team members and inviting the Principal Investigator (EO) to present at research rounds with a recruitment invitation following the rounds. Interested individuals then contacted the study team and a formal email invitation to participate in the study was sent by the Principal Investigator. We also engaged in a process of snowball sampling, whereby study participants recommend additional potential participants; and with permission, these individuals were contacted by email with an invitation to participate in the study.

### Data collection

Data were collected using individual, semi-structured interviews. A semi-structured interview guide was developed by the research team to guide the direction of the interview; however, most interviews were free-flowing conversations, rich with detail and specific examples, thus reflecting the participants’ expertise related to the topic (see Table 1 for sample interview guide). Data were collected by (EO) who has expertise in both neonatal nursing and qualitative research methods. Interviews were conducted in-person (*n* = 2) or by telephone (*n* = 15) and were 30–65 min in length. All interviews were digitally recorded and transcribed verbatim. Field notes were recorded by the researcher following each interview; these included relevant contextual information as well as initial analytic impressions.

**Table 1:** Sample Interview Guide

**Introduction:** In this interview I will ask you questions about your professional experiences caring for young parents with newborns in the NICU	
**Questions**	**Prompts/Probes**
I’d like to start by having you tell me a little about yourself and role	PROBE re: aims of care PROBE re: how are parents referred to your services? PROBE re: how many young parents?
From your perspective as a care provider what are your observations of the challenges experienced by the parents of any age when their baby is admitted to NICU?	PROBE re: how does this affect the parent (physically, mentally etc.)?
From your perspective what are the unique challenges for an adolescent parent with a new baby in the NICU?	PROBE re: how do challenges impact experiences?
What are the challenges for providers caring for young parents and their infants in the NICU?	PROBE re: how do you have to adapt the care/services you provide to a young parent/infant?
What is something you or your organization does well in supporting young parents in the NICU?	PROBE re: are there areas that could be improved PROBE re: additional supports needed
If you could design the best possible program or services to support young parents to successfully navigate NICU admission and transition-home, what would it look like?	PROBE re: what would be helpful? PROBE re: who should be involved in this care? PROBE re: where/how should this support happen?

### Ethical considerations

This study was approved by the Hamilton Integrated Research Ethics Board (HiREB # 5089) with additional approvals obtained from partnering PHUs where requested. Written informed consent was collected from all participants prior to the scheduled interview date, with a verbal confirmation of consent conducted prior to commencement of data generation. Audio recordings were destroyed following transcription and transcripts were anonymized.

### Data analysis

For this study, data collection and analysis progressed concurrently, with analytic memos informing each subsequent interview and contributing to the overall analysis process. Analysis in interpretive description is inductive, and thus began with immersion in the data set [34]. A constant comparative analytic approach was then applied, comparing similarities and differences both within and across cases. Additionally, developing patterns were defined, described, and challenged in analytic memos and a process of diagramming was used to explore relationships between developing patterns [38, 39].

Preliminarily analysis was completed independently by the principal investigator (EO), however, a sample of transcripts were reviewed by the entire research team (MB, AG & SMJ initials). Additionally, developing patterns were discussed as a team throughout the analytic process. To further advance the analytic thinking of the principal investigator and enhance analytic credibility (i.e., peer debriefing), two sample transcripts and initial analytic insights were presented to a qualitative research interest group to which [EO] and [SMJ] are members.

### Rigour

Methods to enhance rigor, informed by interpretive description design methodology, included (a) research questions and methods consistent with the methodological design and for the purpose of informing practice; (b) clear descriptions of analytic strategies used; (c) findings that move beyond a simple description of the phenomenon (i.e., highlighting patterns, relationships, and variation); and (d) findings that are grounded in the data, with exemplar quotes, thus facilitating access to the researcher’s line of reasoning [34]. Additionally, standards were followed for the reporting of qualitative research [40].

## Results

The results of this analysis represent the experiences of 23 providers: 18 registered nurses (RN), three nurse practitioners, and two social workers. Participants worked in either the NICU setting (*n* = 8; level II and III NICUs and neonatal follow-up) or public health (*n* = 13); two participants had experience working in both the NICU and community settings. Those participants from public heath were specialized nurses delivering the evidenced-based Nurse-Family Partnership (NFP) intervention (see description of program in Participants above) and spoke to the experience of supporting their NFP clients throughout an NICU admission (as well as their unique perspective of supporting clients before, during, and after admission given the intervention timeframe < 28 weeks during pregnancy to the child’s second birthday). All participants were women and had at least two years of practice experience; with four providers having over 30 years of clinical expertise (mean, 17 years; standard deviation, 8.9 years; range, 2–31 years).

**Fig. 1:**
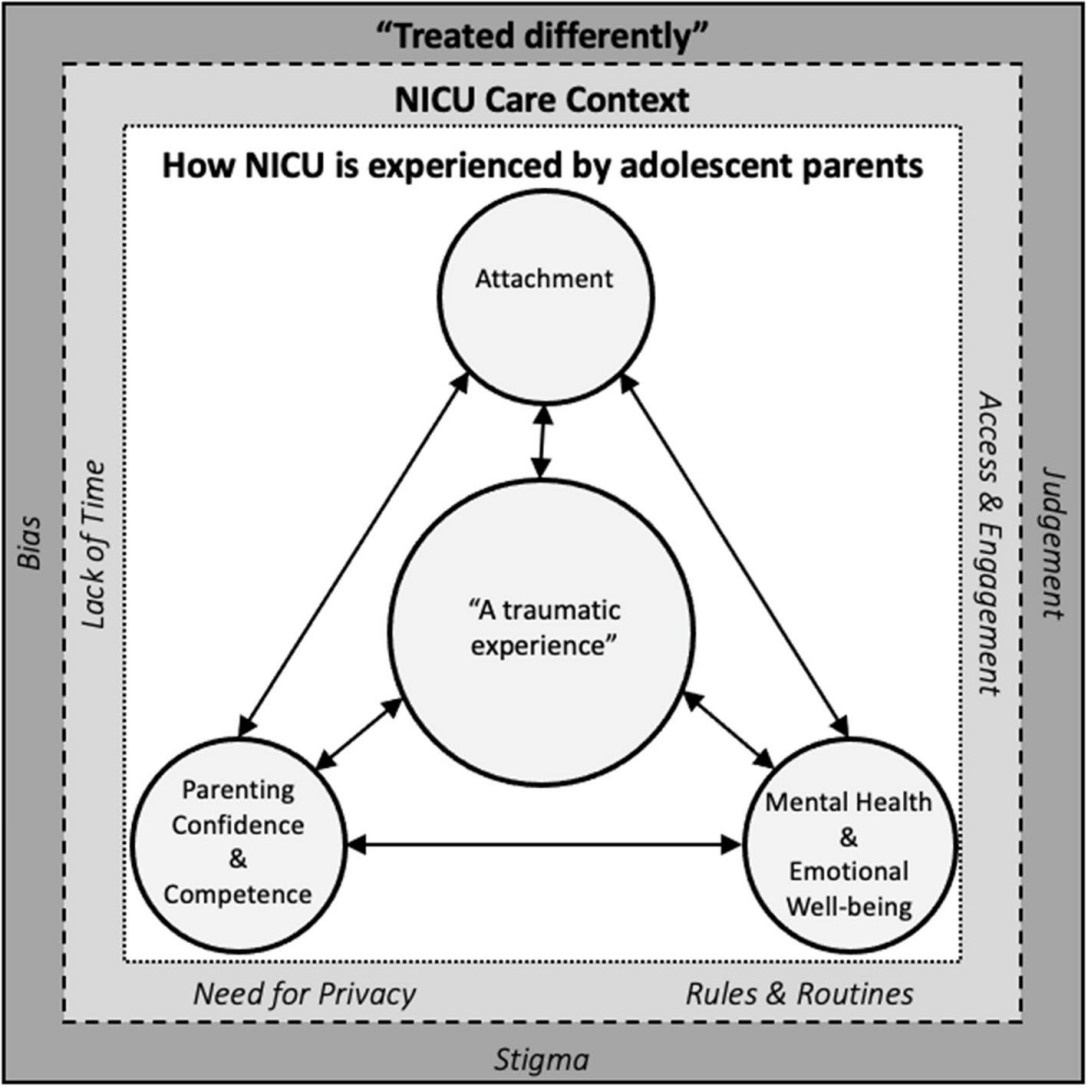
The NICU Care Context and its Influence on the Care and Experiences of Adolescent Parents

Providers interviewed for this study identified that the NICU care context was perceived to influence how care was provided to adolescent parents thus impacting how they experienced the NICU – particularly that having a baby in the NICU was perceived as a traumatic experience for the young adolescent parents they cared for. The providers’ accounts also spoke to the perceived impact of this experience on these young peoples’ lives including mental health and emotional well-being, parent–child attachment, and as they navigated transitions to the parenting role in the NICU. Also present in these providers’ accounts was how they perceived adolescent parents being “treated differently” as influencing the entire NICU experience in that the perceptions of bias, stigma, and judgement by care providers penetrated all aspects of the overall experience. Figure 1 is a visual representation of the findings that demonstrates how each of the patterns resulting from the analysis are interconnected, thus, highlighting the perceived complexity of the NICU context and experience for adolescent parents.

### Influence of NICU care context

From the providers’ perspectives, the NICU environment had a significant impact on how care was provided to and experienced by adolescent parents. These factors included the fast-paced nature of care in the NICU and the lack of time for providers to assess for and then address social or emotional needs that were beyond hands-on care needs of the infant, the lack of privacy available in NICU spaces, the rigidity of rules especially related to infant care and visiting, and the accessibility of the NICU especially related to transportation and out-of-pocket costs.

#### “Time is not fair in the NICU”

Providers described the high degree of critical illness among infants in the NICU environment which required health care professionals to prioritize their time and actions to addressing the mostly physical health needs of the infant. This reality often then meant that less time was available to address other social or emotional needs of the family or any needs outside of “keeping those [on a ventilator] babies alive.” These needs included emotional support for parents, health teaching, and even the interpersonal connection required for developing a therapeutic relationship with the parent and family, as this NICU nurse described:

And time in the NICU is not fair. I have the skill set and the ability to help these moms, but with all of the other constraints and the acuity that's happening, you just you can't do it. And I think that's a really hard spot to be in as a nurse and a provider, because we're not providing these families, especially young families, with the care they deserve.

This lack of adequate time was also described when preparing parents for discharge from the NICU. Despite discharge being perceived as a time of heightened anxiety for parents, with promoting parenting confidence and social emotional support at their peak, appropriate attention to these needs were seen as difficult to prioritize.

#### Privacy

Many providers described limited opportunities to maintain family privacy in the NICU as a factor that contributed to the negative NICU experiences. This decreased privacy faced by parents was seen as impacting the development of trusting relationships between parents and staff, contributing to the emotional toll of the NICU, and eliciting the general feelings of discomfort in the environment. Additionally, a PHN described the impact that witnessing others’ suffering or grief had on their clients:

And I think they often see a lot of other things happen in the NICU that even if their baby's stable another baby they've gotten to know may not be. And so, I think just there's a whole lot of feelings that come with that. The guilt that someone else struggled more in the NICU or just a feeling of fear. They have to get that off their chest and process a little bit.

Private rooms were viewed as the remedy for issues related to privacy, however, communal spaces such as parent lounges and waiting rooms were perceived as potentially “hostile” for younger parents, where there was potential for judgement from other families and visitors.

#### Rules and routines

From the provider perspective, rules and routines in the NICU were experienced by parents as overly rigid and often contributed to restrictions on their level of comfort in the NICU and feelings of exclusion from their infant’s care. Visiting rules seemed to particularly impact young parents and other non-traditional family relationships. Visiting rules varied across the units represented by the providers in this study; for example, one NICU limited visitors to parents and grandparents of the infant, where another did not limit who could visit but how many individuals could visit at a time and when (maximum of 2 individuals, but only parents within certain hours). NICU visiting guidelines were often a reflection of more traditional families (2 parents) or more traditional support patterns for women in the postpartum period (parent and their parents). These rules or guidelines demonstrated a lack understanding of the different types of support systems parents may have outside of more traditional definitions; this was perceived as impacting the adolescent parents specifically:

I know a lot of [adolescent parents] ... their supports aren't necessarily immediate family. So, it may be friends, cousins ... what not. And here in our NICU there are specific visiting guidelines, so only parents and grandparents. So, I have seen that being a bit of a barrier where perhaps a friend is a support person ... exceptions are of course made, but I think that's a potential barrier to some of these girls and not feeling confident or having that assertiveness to say 'this my support ... I don't have a relationship with my mom ..

Rules and routines in the NICU also governed when care was provided (e.g., feedings, diaper changes, etc.) as well as the availability of staff to provide support. For staff, these routines maintained an order in an otherwise chaotic environment. However, when reflecting on how they were received by parents, providers often acknowledged how rules or timing of care could limit parents’ connection with their infant and promote the notion that parents are outside their infant’s circle of care. As this NICU nurse reflected:

And so, I think sometimes there's rigidity around our routines and what we do, which I think can impair that trust, because I think that it sometimes doesn't feel to families like we're really family-centred. I think we say that we are. But in a lot of ways, I'm sure it doesn't feel like that to families.

Tailoring schedules or processes within the NICU to meet parents’ needs was seen as an exception rather than the norm, and parents that did not fall in line with the routine missed out on care or teaching opportunities, conversations with the NICU team, or were labeled as “disengaged.” The challenges with strict routines, especially related to timing of care and ability to engage with their infant, was seen to significantly impact parents with issues accessing the NICU; an issue perceived by providers as common among younger parents.

#### Factors that limit parental access and engagement

Providers recognized that access to the NICU is a perceived challenge among many parents, but especially younger parents or those representing other marginalized groups (e.g., recent immigrant families). Access to reliable or affordable transportation to travel to the hospital on a regular basis was a perceived barrier that was observed to limit parents’ ability to spend regular or extended periods of time with their infant in the NICU. Most NICUs deliver care to families across large geographic regions and therefore many parents need to travel, sometimes significant distances, to be with their infant in the NICU. Younger parents often did not have access to their own vehicle and therefore relied on rides from family members or friends or used public transit. Several providers shared that when this barrier was not recognized by NICU staff as an issue of access, it often became a point of judgement, as this social worker account highlights:

I think travel is a really big one because here in [region] we have one centralized NICU now. And I know staff will say, like, you know, 'this mom ... she hasn't been coming in...', but is she is not able to get in? Does she not have money for a cab? She doesn't drive. She lives 45 minutes away from the hospital. How is she going to get there and how can we support her in getting there? I think that's a really big barrier.

Costs associated with visiting the NICU, such as food and drinks, were also perceived barriers to accessing the NICU that resulted in parents spending less time with their infant; time that could contribute to attachment and development of parenting skills.

While ways to address these more tangible barriers to access, such as food, transportation, and parking, were discussed by providers, only a few recognized that the parent’s perception of the NICU environment as unwelcoming or threatening in general may serve as an invisible barrier, as this NICU nurse described:

Sometimes I think we do see families that maybe don't visit as much as we think that they should. And I think for a lot of families, that can be because they just may not even feel comfortable in the environment. They may not feel like they're welcome there. So, we might see that they're more absent than we would quote “expect them to be” with their infant in the NICU.

This understanding of the more psychological factors to accessing the NICU was seen as critical, especially for those experiencing mental health challenges, past experiences of trauma, and feelings of age-related stigma. Providers also discussed how in some cases, these contextual or environmental factors not only impacted how care was provided but contributed to an overall negative experience of the NICU; with impacts on parent-infant attachment, parenting confidence, and mental health or emotional well-being.

### NICU experience and its potential impact

Providers consistently spoke to the profound impact of the NICU hospitalization for the adolescent parents they cared for. This experience was commonly described as traumatic and was seen to influence attachment, parenting confidence or competence, and mental health or emotional well-being.

#### “A Traumatic experience”

Many of the providers referred to the NICU experience as traumatic or traumatizing for the parents to whom they provided care. Among those who did not specifically label the experience as “traumatic”, they instead described the types of emotional or psychological responses associated with traumatic events – such as fear, anxiety, and grief – among the parents they cared for. One provider reflected on their 35-year career working in the NICU and how few parents were unaffected by the NICU experience – regardless of age or circumstances surrounding the infant’s need for care:

And that's the other thing that I've felt my whole career ... if you have a baby, you remember forever having the baby in an intensive care unit. And so, whether your baby was born really preterm or the baby had transient tachypnea and was not particularly sick ... I think that it is just as traumatic … it doesn't matter how sick you were, forever you will remember that you had a child in the NICU, and that's traumatic.

While the NICU providers recognized the traumatic nature of having one’s infant admitted to the NICU in general, it was typically the PHNs who discussed the importance of the intersection between a stressful stay in the NICU and young parents’ previous experiences of trauma. These intersecting experiences were perceived as contributing to exacerbated levels of stress during an already complex parenting experience. For many of the PHNs in this study, their clients had experienced various forms of trauma and violence across their lifespan including interpersonal and structural racism, child abuse and/or neglect, and intimate partner violence. The fear and uncertainty of the NICU was experienced as another threat to which these young parents had limited or inappropriate strategies to cope, as this PHN described:

A lot of the NFP clients, you know, they've gone through some kind of trauma. So, dealing with all that social piece, and then on top of that if you were to have the NICU experience, depending on how that journey was … is an added stress to their life.

### Impact on attachment

Providers were very aware of the negative impact of separation of parent and infant when the infant’s condition warranted NICU intervention shortly after birth; with both NICU and community providers describing the effects on parental attachment regardless of age. However, adolescent parents were often seen as needing more support to overcome this separation and its potential impact on attachment; including support in establishing an emotional connection with their infant as well as tangible support overcoming situational, social, and economic forces that perpetuate the initial separation following birth as the parent becomes a “visitor” of their infant in the NICU:

I think one unique challenge, because of the forced separation there—it's getting [adolescent parents] to commit to come back for the variety of reasons while baby is admitted ... the importance of them being there as often as they can [especially for] attachment, baby's physical stability and growth and development. So quite often there's a little bit more health teaching that must go into that to encourage them to be there [in the NICU] because I guess just developmentally, they might not be ready to commit that amount of themselves without a lot of extra support.

Additionally, the skill and expertise with which the NICU nurses provided care was also seen as restricting or inhibiting the developing bond between parent and infant, as this PHN observed:

So, I can only imagine ... [the young parent has] to sit there and watch someone else do it [provide infant care] with such confidence. You know, kind of watching the nurses do everything for [their infant] that I can't help but wonder if that impacts their bond or the attachment with their baby from the beginning.

In the NICU providers’ reflections on supporting younger parents, they recognized the unique needs of this group within the NICU context; for example, younger parents were seen to need more teaching related to basic parenting skills to increase attachment. However, practicing in such a way that included parents and supported attachment was often viewed as difficult to operationalize in the NICU context, as this NICU RN questioned “So how do you have time to adequately prepare a young adolescent mom and potentially her partner when you're putting out fires left, right and center?”.

### Impact on parenting confidence and competence

Beyond identifying opportunities to promote attachment, the NICU experience was perceived to impact the development of parenting knowledge and skills that served to support new parents’ confidence and competence in the transition to the parental role. As analysis progressed, a paradox became evident; NICU providers understood the importance of focusing on this role attainment, but had difficulty integrating parents’ learning and needs into the care of their infant in meaningful ways that supported this growth:

I think [there are] a lot of expectations that parents will be able to competently perform tasks like checking the baby's temperature or changing the diaper or calming the baby down. But we don't always step back to think, “Have we really walked a parent through how to do this when their baby is intubated or really sick?” You know, there's a bit of that fear, I think, from parents and I'm not sure we're always good at coaching them through it.

This impact on developing the parental role was viewed as particularly challenging for “the younger ones more than the older [parents].” These challenges were perceived to be related to the busyness of the NICU as well as individual nurses’ comfort and skill partnering with parents to provide care and promote parenting confidence.

Younger parents were also seen as having higher needs with respect to parenting knowledge and skills when compared to adult-age parents. Development of parenting capacity was not seen as a priority in the context of these young parents’ lives as they were experiencing other life challenges such as poverty, mental illness, and experiences of poor parenting practices from their own childhood. Supporting this level of parenting need within the NICU was seen as a consistent challenge across NICU providers and something that could only be done in “an ideal world, if staffing was wonderful.” Ultimately, this was problematic because, parenting skills and parents’ abilities to care for their infants were often the criteria by which providers judged the competency of a parent within the NICU, as this NICU nurse highlighted:

[Adolescent parents] are placed in the NICU environment. It's very sterile. It's very regimented in what happens when … and [adolescent parents] don't necessarily know what to do ... it's reflected poorly upon them in charting, and to the physicians.

The feelings of detachment from their infant, lack of confidence in the parenting role, and feelings of judgement based on parenting competence contribute to a vulnerable state for young parents in the NICU and likely contribute to the NICU being experienced as traumatic.

### Impact on mental health or emotional well-being

Mental health or emotional well-being has a similar relationship within the overall NICU experience; such that the experience is perceived to affect young parents’ mental health and pre-existing mental health challenges can contribute to how hospitalization in the NICU is experienced. Young parents’ experiences of pre-existing mental health issues or the onset of perinatal mood disorders were described as a particular challenge by providers in the NICU. Social workers in the NICU saw supporting mental health or emotional well-being as a significant component of their role and nurses relied on social workers to provide this type of support for families due to the challenge of prioritizing social-emotional care for parents and the bio-physical needs of the infant. The NICU was also perceived to exacerbate experiences of mental health challenges for parents of all ages, something NICU nurses felt ill-equipped to manage, as this NICU nurse describes:

Yeah, I think that's not just the young ones, I think that's for everybody. And the push is not just the mental health issues, it's mental health issues when, you know, you've just had a baby, you're stressed and that kind of thing. It's just heightens everything. I honestly don't think we do great with that … I think one of the things we probably don't do as well as we could is supporting moms with mental health issues.

Many of the PHNs in this study were employed in programs that start working with clients early in pregnancy and provide regular support until the infant is two years of age; with weekly home visits in the initial postpartum period. These PHN participants noted a need for emotional support in their young clients when infants were admitted to the NICU; often continuing their “home visits” in the NICU to provide this support to clients. Even parents that were seen as coping well with other NICU related challenges (e.g., attachment and parenting) were often seen as struggling emotionally, as this PHN described:

This mom did so well in the hospital. I can't even think of something that was really challenging except for what was going on emotionally. So, I did long visits sometimes at the hospital with her. But I was mostly supportive, listening and, you know, supportive guidance with her and reassuring her.

When pre-existing mental health challenges or the emotional impact of the NICU was not recognized or addressed, parents often received the label “difficult,” thus creating tensions with the care team and a breakdown in the therapeutic relationships between providers and parents. Mental health challenges and perceptions of judgement were also perceived as contributing to ineffective coping upon discharge, prolonged emotional distress, and the overall assessment of the NICU experience as traumatic.

Physical and emotional separation leading to poor parent-infant attachment, difficulties establishing parenting confidence and competence, and challenges with mental health or emotional well-being including potential for (re)traumatization is likely common, to a certain degree, among all parents with infants in the NICU – with differences and distinctions observed across all demographic variables – not simply age. Even the providers in this study, who described scenarios and circumstances where the NICU experience was more challenging for younger parents, noted that this was not always the rule without variation.

#### “Treated differently”

One common thread throughout the providers’ accounts was that younger parents’ experiences of stigma or judgement – whether actual or perceived – served to underscore the entire experience of the NICU for this group. Providers from both the NICU and community reflected on examples of young parents being treated differently, and more negatively, compared to other parents due to ageism. As this nurse who worked both in the NICU and as a PHN described:

I've found as well ... just kind of the treatment of some of the younger girls is not necessarily the same as if they were a 30-year-old, educated individual ... [they are judged on their] outward appearance, involvement on their phones, the way they even interact with the babies.

Providers often observed implicit bias against younger parents during staff handover or other staff-to-staff interactions with a common example of such bias being the qualification of a young parent’s progress or success as being good “for their age.” Providers also felt bias was present in nonverbal behaviours of staff or the perceived warmth offered some parents versus those that appeared “rough” or “edgy.” From the providers’ accounts, it was also clear that young parents were often aware of this staff bias and/or differences in treatment based on their age as this NICU social worker pointed out “I know [they perceive bias] because [adolescent parents] told me, and they're like 'oh this is because I'm this age.”

This finding of stigma or bias against younger parents was not viewed as a NICU exclusive problem; but a general societal judgement that parenthood should look a certain way. These societal views, however, impacted the way young parents experienced hospitalization of their infant in the NICU, thus compounding the aforementioned effects of the NICU environment and leading to an NICU experience that is considered traumatic.

## Discussion

To our knowledge this is the first study to ask providers caring for adolescent parents in the NICU about how the NICU context influences care provision as well as how they perceive this to impact the overall experiences of adolescent parents with an infant in the NICU. In this study we were able to capitalize on the abundant knowledge and skill of these expert providers, who offered insight into the complexity of caring for adolescent parents in the NICU. Based on the findings of this study, we understand that the NICU care context can serve as a significant barrier to providers delivering the type of care that contributes to positive outcomes for the adolescent parent/infant dyad (e.g., parent-infant attachment, parenting confidence). These barriers included the rules and routines in the NICU, lack of time and privacy, challenges to access and engagement in the NICU environment, as well as perceptions that adolescent parents being “treated differently”. Central to these findings was that providers in the NICU often had the knowledge and skills required to provide care that could limit negative impacts; however, contextual factors limited this ability especially when the complexity or needs of the parent-infant dyad increased – such is the case with adolescent parents. To mitigate the potential negative impact of the NICU experience on the adolescent parenting journey, findings suggest that tailored approaches to caring for adolescent parents in the NICU are required including enhancements to the NICU model of care and core skill set of NICU providers, collaboration with providers outside of the traditional NICU care team, as well as the adoption of atraumatic care approaches.

### Expanding the NICU interprofessional team

Expanding the NICU interprofessional team to include members that are skilled in addressing complex social, emotional, and psychological needs could serve all parents experiencing the NICU better – but particularly for adolescent parents who have increased prevalence of mental illness antenatally [15], a higher prevalence of postpartum depression [41], and who experience social, economic, and health inequities [42]. NICU providers in this study, were experts in working as part of an interprofessional team and often relied on one another’s knowledge and expertise to care for infants and families in the NICU. For example, nurses relied on the expertise of social workers to address social factors when they arose. However, supporting parents’ mental health and emotional well-being remained challenging in the NICU care context. The mental health impacts experienced by parents, both in the NICU and beyond discharge, have been extensively documented [4, 43] and, based on our findings, were also perceived as a significant aspect of adolescent parents’ NICU experience. Building on the interdisciplinary strengths of the NICU, additional layers of emotional support for parents, beyond nurses and social workers, are warranted. Proposed best practices for supporting the emotional well-being of parents while in the NICU and mitigating the potentially traumatic effects of the NICU experience includes incorporating mental health professionals (psychologists, psychiatrists, and psychiatric nurses) as part of the NICU interprofessional team [43]. These mental health professionals would be responsible for providing emotional support, screening for emotional distress, education, and psychotherapy for families. These professionals could also offer educational and emotional support for the NICU health care staff; as moral distress has become relatively ubiquitous with among NICU practitioners [35].

Beyond mental health practitioners, the findings of this study point to the potential role that PHNs can play in supporting parents in the NICU. Many public health programs aimed at supporting healthy families focus on facilitating early attachment between parent and infant, teaching positive parenting strategies, navigating health and social services, and promoting healthy growth and development among infants and children [44]. Additionally, public health nurses working with families often have an advanced skill set related to recognizing and responding to intimate partner violence [45], supporting maternal mental health [46], and enhancing parenting capacity to mitigate risk of child maltreatment [47]. Recognizing PHNs as experts in these areas and integrating them into the NICU interprofessional team could support parents who have greater needs for developing parenting capacity and confidence as well as those who require additional layers of social and emotional support. The additional benefit integrating PHNs within the NICU interprofessional team would be the anticipatory guidance and continuity of care that these nurses could provide as parents transition home from the NICU; an important ingredient in achieving healthy growth and development post-NICU. However, NICU and public health nursing collaboration remains relatively unexplored and could be a promising avenue of future research [48, 49]. Expanding the interprofessional team can also support the lack of time perceived as a barrier to providing adequate care to more complex families in the NICU including adolescent parents.

### Trauma- and violence-informed care in the NICU

Despite the potential of additional supportive roles within the NICU, the stigma and judgement encountered by adolescent parents as they interact with the health care system, including in the NICU as our analysis suggests, may contravene any benefit. This age-related bias and/or stigma noted by providers in this study can be corroborated by adolescent parents themselves, with age-related discrimination emerging as a strong theme in both interviews and surveys conducted with adolescent parents in NICUs across the United Kingdom [9]. Parenthood can be a positive transformative period in the lives of young people; however, this potential can be rendered ineffective in the presence of stigmatizing interactions and low expectations from providers – such as those encountered in the NICU [50]. Adopting the principles of trauma- and violence-informed care (TVIC) within the NICU could reduce feelings of judgement or discrimination, support the establishment of strong therapeutic relationships between parents and staff, and limit the traumatising effect of the NICU experience [51, 52].

A ‘trauma-informed’ approach refers to the delivery of health, social, mental, and behavioural services, that accounts for possible experiences of trauma [53]. Expanding on this concept, trauma- and violence-informed care draws attention to forms of interpersonal and structural violence that individuals may experience [54]. The inclusion of structural violence is important because social structures within the NICU, such as visiting guidelines or transportation requirements, can reinforce inequities resulting in an uneven distribution of resources that could eventually lead to harm for the parent and/or their  infant [55].

There are four, inter-related principles of a trauma- and violence-informed care approach which could be applied within the NICU: (1) understand trauma and violence on peoples’ lives and behaviours; (2) create emotionally and physically safe environments; (3) foster opportunities for choice, collaboration, and connection; and (4) provide a strengths-based and capacity building approach to support client coping and resilience [54]. Adopting a trauma- and violence-informed care lens in the context of the NICU – where providers understand the life circumstances of the parents they care for – would help to acknowledge and reduce implicit bias toward adolescent parents displayed by staff and the stigmatization experienced by young parents. This approach could also lead to a recognition of the traumatizing effects of the NICU environment as well as efforts towards mitigation of these impacts; therefore, helping reduce harm related to re-traumatization from past exposure to violence and/or Adverse Childhood Experiences (ACEs) [12, 14]. Additionally, strengths-based approaches or care would aim to see and understand the whole family – parent/infant and support persons – with the goal of understanding what is functioning well and what supports are needed to optimise functioning [56]. This approach to care, that is non-judgemental and tailored to each parent/infant’s unique circumstances could see the formation of trusting relationships between NICU staff and adolescent parents, leading to engagement in care and overall parenting confidence moving forward [57].

Adopting universal trauma precautions such as trauma- and violence-informed care, can improve system responses for everyone – not just those with trauma histories, as staff are equipped, and organizations are more responsive to the needs of all people [54]. Integrating trauma- and violence-informed care principles in the NICU could reduce the influence that the environment has on the NICU being experienced as traumatic (e.g., attention to issues of privacy and addressing rules and routines that represent violent structures). Additionally, equipping NICU nurses and staff with trauma- and violence-informed knowledge and opportunities for skill development, like public health nurses working within parent/infant programs, could lead to strong therapeutic relationships between NICU staff and parents that are necessary to mitigate the impact of the NICU experience and support maternal-infant attachment, parenting confidence and competence, and maternal mental health and emotional well-being. Specific examples of trauma- and violence-informed care principles in action in the NICU are outlined in Table 2. Public health nurses working within parent/infant programs are trained in trauma- and violence-informed care.

**Table 2:** Actions Supporting Trauma- and Violence-Informed Care (TVIC) Principles in the NICU

TVIC Principles	Actions supporting Trauma- and Violence-Informed Care in the NICU
Understand trauma and violence on peoples’ lives and behaviours	• Provide staff education on trauma, violence, and its effects with a specific focus on the families and communities the NICU serves • Provide staff education related to the principles of trauma- and violence-informed care • Promote staff understanding of the parent/family experience of the NICU through sharing resources, workshops, and listening to the voices of parents
Create emotionally and physically safe environments	• Ensure welcoming and comfortable physical environments with an emphasis on both physical and emotional safety • Perform a trauma walk-through of the NICU and surrounding spaces • Provide access to food, water, and basic personal care items for parents in the NICU • Review policies and procedures to ensure alignment with trauma- and violence-informed care principles
Foster opportunities for choice, collaboration, and connection	• Support parents’ choice in determining their support person in the NICU • Create routines based on parents’ preferences where possible rather than unit/provider preference (e.g., care routines, rounds) • Encourage models of care that focus on hands-on care by parents with support by nurses and NICU staff
Provide a strengths-based and capacity building approach to support client coping and resilience	• Support parents in recognizing their own strengths and self-care strategies • Provide sufficient time and resources to support meaningful engagement between NICU staff and parents

### Limitations

While the findings of this study provide a rich understanding of the NICU care context for adolescent parents from providers’ perspectives, to develop and extend what was learned from this study, the experiences of young parents need to be investigated. This work is ongoing by the authors but was delayed by the COVID-19 global pandemic. Providers in this study represent both the NICU and community setting, this is an important source of triangulation given the diverse perspectives of the NICU, however, the inclusion of other providers, beyond nurses and social workers, could further enhance analysis. Additionally, nursing workforce challenges and health human resource shortages may have influenced providers’ accounts of the NICU context. However, these were not explicitly addressed in this study and could be the topic of future exploration.

## Conclusion

This analysis presents critical insights into the NICU care context and how it influences care provision for adolescent parents as well as their NICU experiences. This understanding serves as an important foundation for improving care for adolescent parents both in the NICU and as they transition to home and community. Based on the perceived traumatic nature of the NICU experience for many parents, NICU team members can adopt trauma- and violence-informed care principles to reduce harm experienced by parents, especially young parents, while providing safe and responsive care to all NICU families. In efforts to support parenting competence and confidence, attachment, and emotional well-being of young parents in the NICU, collaborations between NICU nurses and public health nurses as well as the incorporation of mental health care providers on the NICU team should be explored.

## Data Availability

The data analyzed during the current study are not publicly available due to confidentiality of the participants but are available from the corresponding author on reasonable request.

## Abbreviations

ACE: Adverse childhood experience
FCC: Family centred care
NFP: Nurse-family partnership
NICU: Neonatal intensive care unit
PHU: Public health unit
PHN: Public health nurse
TVIC: Trauma- and violence-informed Care
